# The Impact of Cognitive Anxiety and the Rating of Pain on Care Processes in a Vigilance Task: The Important Part Played by Age

**DOI:** 10.1155/2020/3204720

**Published:** 2020-04-20

**Authors:** Luis Pinel, Miguel A. Perez-Nieto, Marta Redondo, Luis Rodríguez-Rodríguez, Leticia L. Mateos

**Affiliations:** ^1^Faculty of Education and Health, Camilo José Cela University, Madrid, Spain; ^2^Rheumatology Service, Hospital Clínico Universitario San Carlos, Madrid, Spain

## Abstract

Chronic pain is a serious public health problem that has grown exponentially in recent years, which is why it has received the attention of numerous researchers. Most of the studies in the field of chronic pain have focused on care as a mediating variable on the perception of painful stimuli and emotions. Nevertheless, there are very few studies that have gone in the opposite direction. This study's aim is therefore to analyse the impact of emotional variables (anxiety and depression), the rating of pain, and age on vigilance processes in a sample of patients with chronic pain. To do so, the attentional performance of a cohort of 52 patients with chronic pain was measured through the use of a modified dot-probe task. Furthermore, all the participants were evaluated using the following self-report measures: Beck's Depression Inventory-II (BDI-II), the McGill Pain Questionnaire, and the Pain Anxiety Symptoms Scale-20 (PASS-20). Stepwise multiple linear regression analysis revealed a significant negative correlation between the pain rating index and the number of mistakes the participants made during the attention test. There was also a positive and significant correlation with age and another negative and significant correlation with cognitive anxiety regarding the overall performance times during the undertaking of the experimental task. These results point to the importance of a more in-depth understanding of the impact that the emotional variables and other variables such as age have on attentional processes and the rating of pain. Finally, the discussion focuses on the implications these results could have for clinical practice or for future research studies in this field.

## 1. Introduction

Chronic pain has become a serious public health problem that has grown exponentially in recent years, becoming the most common symptom among patients attending hospital services [1]. This is due not only to its high rates of prevalence [2], but also to the impact this health problem has on a patient's wellbeing, their family, healthcare costs, the loss of productivity at work, and the high socioeconomic costs for the health system (both direct and indirect) [3, 4].

Today, its conceptualisation is understood based on “Gate Control” theory [5], which permits an approach to the concept of pain from a multidimensional model that not only explains its sensorial dimension, but also its cognitive and emotional dimension [6]. This has played a vital role in understanding the contribution and importance of psychological variables in the perception of pain. Most scholars therefore coincide in noting that psychological factors such as anxiety, stress, depression, and anger play a key part in both perceiving and coping with pain, which is why it tends to be a priority goal in most interventions in the field [7–14].

Along these lines, although most researchers in the field have focused on care in their studies as a mediating variable on modulation in the perception of painful stimuli [15–20], we also have evidence to show that high levels of anxiety regarding pain makes patients more mindful of the physical symptoms; in other words, it prompts adopting a state of hypervigilance towards pain or towards any painful stimulus [14]. This hypervigilance towards pain has consistently been associated with higher levels of sensitivity and the perception of pair, greater physical disability, a mistaken interpretation of its symptoms, higher levels of catastrophisation, and a worse behavioural adjustment to the illness [21–24], whereby other variables might also have an influence on the processes of treating and caring for these patients.

For example, we now know that pain becomes more prevalent with age [25, 26]. There are scholars that refer to age as a variable that could modify the rating of pain and alter the cognitive performance in patients with chronic pain [27–29]. Likewise, some studies support the evidence that age correlates with a weaker performance in attention tasks among these patients [30, 31]. It may therefore be described as a variable that needs to be taken into account in the relationship between the rating of pain and the cognitive function, although there is clearly a paucity of studies that have addressed this issue.

The handling of attention is, therefore, a vital component and tends to be a priority objective in traditional interventions, such as Cognitive Behavioural Therapy (CBT) [13], which is now widely used in cases of chronic pain [10, 32, 33]. To do so, the therapy uses distraction as a natural coping strategy for controlling bouts of pain [34]. Distraction strategies are based on the notion that focusing one's attention on stimuli that are unrelated to pain in the immediate surroundings reduces the perception of pain and, therefore, produces analgesia [35]. In turn, there are numerous studies that have found evidence in favour of distraction [10, 36–41]. Nevertheless, despite the rationale and pragmatic nature of these interventions, many scholars report that distraction techniques may not be as equally effective for all patients and that the results of the clinical experiments on their effectiveness are not wholly conclusive [42–46].

At the same time, other theoretical models have emerged that maintain the fact that the problem lies precisely in avoidance [47, 48], which impedes a conscious and elaborative processing of the stimulation being avoided (e.g., painful stimuli, catastrophic thoughts, and emotions) [49]. According to this model [50], the fear related to pain and catastrophic thoughts could lead to a misinterpretation of the symptoms of pain as potentially threatening [45, 51, 52], thereby increasing vigilance towards stimuli related to it [45] and facilitating the avoidance response [53]. Along these same lines, there are many instances of research that have consciously related a fear of pain to a low mood state associated with a decrease in reinforcing activities and an increase in the disability of people with chronic pain [37, 48, 54–61].

In response to the obvious importance of the attentional care of these patients and the significant role that avoidance plays, recent years have witnessed the increased popularity of mindfulness-based interventions (MBIs) for addressing these variables. These interventions are based on a series of skills whose origin lies in ancient Buddhist practices adapted to the western context [62]. MBIs enable an individual to face pain rather than avoiding it and being aware of the thoughts, emotions and physical, and emotional sensations involved in its rating [63, 64]. The overriding objective that the components of these interventions pursue is the self-regulation of attention, which includes processes such as sustained attention, the change in attentional focus, and the inhibition of the elaborative process [65]. Likewise, MBIs lead to a significant functional improvement in patients with chronic pain, less hypervigilance, and an enhanced emotional performance [64, 66–68]. Nevertheless, although the efficacy of these interventions has been proven by research, and acceptance-based interventions are more coherent at theoretical level, by ending with avoidance and putting the individual in touch with their private experience [69], recent studies of meta-analytical review show that these results are not consistent when finding differences in terms of efficacy between MBIs and CBT [70, 71]. These data prompt the need to continue furthering our understanding of attention and other psychological variables (e.g., fear of pain, catastrophisation, anxiety, depression, or pain itself) that may help to explain the variability of these results applied to clinical practice.

Given the aforementioned paucity of studies that have investigated this topic, the aim of this research is to analyse the relationship between depression, the pain rating index (PRI), cognitive anxiety, and age, as well as its ability to predict a better performance in an attention task based on the dot-probe test, whose characteristics may well make it the most suitable way of measuring the number of mistakes and total performance times in relation to the study variables [72–76], basing ourselves accordingly on the theoretical model proposed by Bishop et al. [65]. This will enable us to go some way to clarifying the possible implications this information might have for clinical practice in terms of the attentional components that dot-probe tests measure (change of attentional focus and the inhibition of the elaborative process) and the said psychological variables.

## 2. Materials and Methods

### 2.1. Participants

The final sample consisted of fifty-two adults with different diagnoses of illnesses involving chronic pain. Most of the participants had been diagnosed with rheumatoid arthritis. The average age was 54.79. The inclusion criteria were that the patients had received a diagnosis of rheumatoid arthritis, fibromyalgia, or low back pain, they were over the age of 18, and they took part on a voluntary basis. Some of the participants were patients undergoing treatment at the Department of Rheumatology at the Hospital Clínico San Carlos in Madrid (Spain), while others were members of the Madrid Association of Patients with Rheumatoid Arthritis (AMAPAR, in its Spanish acronym).

### 2.2. Assessment Instruments

#### 2.2.1. Self-Report Measures

 
*BDI-II*. Beck's Depression Inventory-II–by Beck et al. [77], in its abbreviated form adapted into Spanish by Sanz et al. [78]. This self-report instrument consists of 21 items that, with good scores in terms of validity and reliability, use a four-point Likert-type scale to quantify the seriousness of the depressive symptomology over the two previous weeks. Our sample recorded excellent levels of reliability and internal consistency (Cronbach's alpha = 0.854), following the criteria proposed by Prieto and Muñiz [79].
 
*McGill*. The McGill Pain Questionnaire (Melzack) [80], in its Spanish version by Lázaro et al. [81]. The questionnaire consists of a list of 19 descriptors that report on how each patient rates their pain. It evaluates both quantitative and qualitative aspects of pain, such as location, quality, temporal properties, and intensity. In this study, this instrument has recorded appropriate levels of reliability and internal consistency (Cronbach's alpha), specifically 0.738, which were suitable according to the criteria of Prieto et al. [79].
 
*PASS-20*. The Pain Anxiety Symptoms Scale-20 (McCracken et al.) [82]. This instrument explores components involving anxiety towards pain: fear, escape/avoidance, and physiological and cognitive anxiety. The scale contains 20 items with a Likert-type answer option ranging from 1 (never) to 5 (always). Our sample again recorded good levels of reliability and internal consistency (Cronbach's alpha = 0.881) according to Prieto et al. [79].
- *Stimulus words*. A set of Spanish words related to pain adapted from Haggman et al. [83]. Some of the words used involve a rigorous selection of emotional and sensorial words from the McGill Pain Questionnaire, along with others that have been successfully used in prior studies [84]. Finally, the neutral words (unrelated to pain) were adapted from the list used by Haggman et al. [83]. The overall aim was to create the list used in the experimental task (see Table 1).

### 2.3. Dot-Probe Task

This involved a test based on the Posner paradigm [85]. The ad hoc test administered for this study is a modified version of the visual dot-probe task [74, 84], consisting in total of two sets of 32 words, one related to pain and one neutral. A dark screen displayed a fixation point for 500 ms and then immediately showed two words, one on the left and another on the right of the screen, and the individual had to indicate the neutral word. Once the choice had been made, by pressing the letters “w” (left) and “n” (right) on the keyboard, the neutral word was replaced by another one until the whole series had appeared. The participants were unaware of where the fixation point was going to appear in each one of the rounds. It is assumed in the tests that the cross coincided with the appearance of the neutral stimulus, which would facilitate the answer, and therefore the reaction times should decrease. By contrast, if the individual's attention was focused on the word related to pain, it was assumed that the response times should increase, particularly in those patients whose psychological variables so permitted.

### 2.4. Procedure

**Figure 1:**
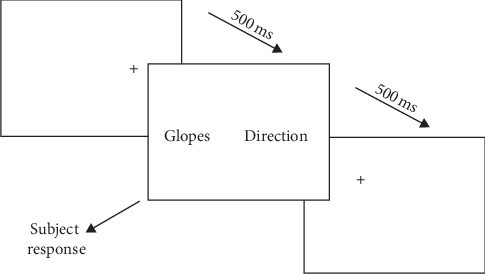
Images of the first three slides in the dot-probe experiment.

The sample's participants were assessed in the Department of Rheumatology at the Hospital Clínico San Carlos in Madrid and at AMAPAR. After verifying that the patients met the criteria for inclusion in the study, they were asked to sign an informed consent form. The assessment was held in a single session that lasted approximately one hour and was always conducted by the same evaluator. The necessary instructions were then given for the dot-probe test. The participants were instructed to press “w” on the keyboard if the neutral word appeared on the left of the screen and “n” if it was displayed on the right. The opportunity was then taken to hold a practice run with a total of 15 neutral words. This was then followed by the modified visual dot-probe test itself with a total of 32 words, some of which were related to pain and others were neutral, as already mentioned (see Figure 1). Finally, the assessment session began and the participants were asked to complete the self-report measures that had previously been approved by the Ethics Committee at the Hospital Clínico San Carlos.

### 2.5. Data Analysis

The data were coded and analysed with version 25.0 of the SPSS statistical package for Windows. The study's initial objectives were achieved by means of a stepwise multiple linear regression analysis. The independent or predictor variables used were the scale of symptoms of anxiety toward pain, the PRI, emotional variables (depression), and the participants' age, while the dependent variables were the number of mistakes made and the total performance time measured in milliseconds, evaluated with the attention test.

## 3. Results

Participants' demographics, relevant clinical variables, and questionnaire scores are provided in Table 2. A total of 52 patients with chronic pain were evaluated, as previously stated, with the majority (94.2%; *n* = 52) being diagnosed with rheumatoid arthritis, a couple of cases (3.8%; *n* = (2) of low back pain, and one case (1.9%; *n* = (1) of fibromyalgia. Concerning the time elapsed since the first medical diagnosis, for 3.8%, it was less than a year, for 9.6% less than three years, for 3.8% less than five years, for 26.9% between five and ten years, and for 55.8% more than ten years.

About 25% of the sample were males and 75% females, with ages ranging between 27 and 77. The average age was 54.79, and the standard deviation was ± 10.93. The majority were married (53.8%) or single (25%), and the rest were widowed (5.8%), divorced (7.7%), or separated (7.7%). About a quarter (25%) of the participants had attained a tertiary education, the vast majority (71,3%) attended general education, high school, or vocational school, and the rest had unregulated studies (3.8%). At the time of the study, most participants were employed (42.3%), only a few (11.5%) were unemployed, a quarter of the participants were retired (25%), and the rest were students (3.8%) or had other employment status (17.3%). The majority of participants reported medium incomes (65.4%), some reported low income (23.1%), and just a few were indiviuals of higher income (11.5%).

As regards the questionnaires, the mean BDI score of the sample was 4.21 ± 4.5 SD, the mean of the scales of the pain rating index measured through the McGill Pain Questionnaire (PRI) was 22.19 ± 8.3 SD, and the mean of the scale of cognitive anxiety towards pain measured by PASS-20 was 12.96 ± 4.4.

In keeping with the study's main objective, our aim was to analyse the relationship between depression, the PRI, cognitive anxiety, age, and the performance in the experimental task based on dot-probe. This involved conducting two stepwise linear regression analyses for each one of the dependent variables.

In the first one, the dependent variable was the number of mistakes the individuals made in the attentional test, while the independent variables were depression, cognitive anxiety, the five scales of the PRI, and age. Verification of the assumptions required for undertaking this kind of analysis was made beforehand, and the display of graphs confirmed that there were no problems related to normality, homoscedasticity, or linearity, collinearity, as indicated by the tolerance values (<0.10) and the variance inflation factor (VIF > 10), or independence (*Durbin-Watson* = 2.128), all of which indicate that the data were suitable for this analysis. Accordingly, the regression analyses conducted for the number of mistakes made by the participants produced a statistically significant model (*F* = 4.430, *p* ≤ 0.05), which explained 6.3% of the variance (adjusted R-squared = 0.63) and produced a single predictor. The total PRI was obtained through the McGill Pain Questionnaire (*Beta* = −0.285, *p* ≤ 0.05) (see Table 3), whereby this variable predicts more mistakes in the experimental tasks. None of the other independent variables were significant.

In the second case, the same analyses were conducted, only that this time the dependent variable was the total time the participants took to complete the experimental task (measured in milliseconds) and the independent variables were depression, the overall PRI, the subscale of cognitive anxiety towards pain, and the patients' age. The aim on this occasion was to discover whether any of these variables allowed predicting a better performance in the attentional task.  Table 4 shows that two statistically significant models were obtained; a first model helped to explain 15% of the variance associated with the total time needed to complete the task (adjusted R-squared = 0.158) and was statistically significant (*F* = 10.605, *p* ≤ 0.05). This model's sole predictor was the patients' age measured in years (*Beta* = −0.418, *p* ≤ 0.05). By contrast, Table 4 also reflects a second analysis that increased significantly (*F* change = 6.467,*p* ≤ 0.01), raising the explained variance by 9.6% (change of *R*^2^ = 0.096). This second model added age as another predictor and was statistically significant (*F* = 9.116,*p* ≤ 0.01). This new model would explain 24.1% of the variance associated with total performance time (adjusted R-squared = 0.241) and included cognitive anxiety as a significant predictor (*Beta* = −0.310, *p* ≤ 0.05) of the response times of those participating in the study.

## 4. Discussion

The results reflect a negative relationship between the PRI and the mistakes made during the performance of dot-probe. This is perhaps the study's most surprising finding, whereby if we base ourselves on the results, the greater expression of pain (through the numbers assigned to McGill's descriptive terms) is associated with making fewer mistakes in the test. This relationship is small (−0.28), according to Cohen's criteria [86]. These findings contradict those reported in the scientific literature, with many studies stating that people with chronic pain have a selective memory regarding pain-related words [87]. The widely held notion is that these patients selectively process information on their illness [88–93]. As their rating of pain increases, they tend to select the information related to it, they are more hypervigilant in response to this stimulation, and they would therefore find it more difficult to disengage their attention from it [94–97]. Nonetheless, we have already noted that detecting the attentional bias is not consistent in all the studies [73, 98–100]. In this vein, our results do not permit us to confirm the presence of this bias. One possible explanation for these results could lie in the processes of learning to tolerate pain. The repetitive and continuous exposure to a painful stimulation helps to reduce the rating of pain and the responses associated with it [101–104]. This means that if patients are subjectively accustomed to pain, they may not only make fewer mistakes but may also have more clues for correctly identifying the pain-related word and identifying it more easily.

As regards age as a predictor variable over the total time taken to perform the attentional task, the results show a direct and significant relationship between both variables. According to Cohen [86], furthermore, this relationship would attain an average size (0.42). These data suggest that the older a person is, the longer the time required to complete an experimental task is. A possible explanation for these results would be that pain, given its stimulating characteristics, competes for the attentional resources that the individual has available [37, 105], whereby the average reaction times in these patients should be longer. In turn, and as noted earlier, it is important to remember that the prevalence of pain increases with age [25, 26], which is why many scholars have found a direct relationship between this variable and a weaker cognitive function in patients with chronic pain [27–29]. It is therefore important to consider the particularities of attentional and perceptive processes in very old people. Some scholars contend accordingly that more time is required in old aged people to process any stimulus [106] and that the ability to remain vigilant in a demanding task will also be affected [107]. The results reported here would thus be wholly consistent with the current scientific literature. Nevertheless, these results highlight the importance of further research for better understanding the role of age and its implications for attentional processes among these patients, as there is a noticeable lack of studies on this matter.

In turn, cognitive anxiety in our study has proven to be a predictor of a shorter response time in the performance during the attention test. Again, according to Cohen's criteria [86], the negative relationship is small (−0.34). There is currently an abundance of evidence to show that anxiety has an influence on the performance of a particular task [108]. Although it is generally accepted that emotional processes have a negative impact, generating an additional cost in resources, there are scholars that contend that preoccupation or concern, rather than emotion in the strictest sense, prompts this effect [109, 110]. In this same vein, we encounter processing efficiency theory [108, 111, 112]. According to these authors, anxiety allows for a more diligent assignment of resources designed to improve the processing of information and performance in demanding tasks. Likewise, greater anxiety can increase the attentional resources focusing on the stimuli that pose a threat in these kinds of individuals [113, 114], and what is more, they have a greater capability in vigilance tasks [115]. In view of this, it is no surprise that greater cognitive anxiety regarding pain is associated with a faster completion of the task in our sample. Nevertheless, more experimental studies are required for a closer understanding of this relationship in patients with chronic pain.

Finally, this study has been unable to find a relationship between depression and the average time the participants spent on the dot-probe task, despite being consistent with the theory. Many researchers contend that sadness significantly reduces the speed of processing information in the field of pain [116]. Nevertheless, this may be attributed to issues of sensitivity involving the questionnaire chosen for evaluating this variable [78].

In short, it is important to know the impact these results might have on clinical practice or future research. Our findings here suggest that BMIs are not effective for all kinds of pain-affected patients. Age has been revealed as a major variable for predicting a worse performance in terms of executive functions. The idea that age is linked to a worse attentional and memory performance has been supported by several scholars in the field [27–29], as commented earlier. This leads us to consider that older people could benefit more from the practice of mindfulness if they have previously undergone some kind of intervention based on CBT; more specifically, cognitive restructuring may help them to adapt and work on attentional components to make it subsequently easier to apply other procedures. In turn, variables such as cognitive anxiety or a higher PRI among these patients seem to be affecting their ability in terms of vigilance. This means that, during a visit to the doctor, it is important to assess those aspects a priori to guide the treatment with these people. As a recommendation regarding these patients, a traditional intervention based on CBT may be more useful for addressing cognitive mistakes and promoting an active approach to coping with the illness [10], as there is evidence to show that cognitions and beliefs have a huge influence on these patients' coping strategies and, in turn, on the rating of pain [53, 117]. In terms of future research, and due to the scarcity of studies on the matter, it would be expedient to repeat studies of this kind, albeit preselecting the participants according to these variables. This would allow observing the differences between different groups, for example, between very old and young people or profiles of high anxiety and those with low cognitive anxiety.

These suggestions, as well as this research's results and conclusions, should be considered within the context of certain limitations. Firstly, this research involved a small sample of patients. This limitation affects mainly the type of analysis undertaken and the processing of the data gathered, whereby it would have been more pertinent to have had more participants in order to enlarge the scope of the results' impact and their reliability. In turn, a very uniform sample was selected in which most of the participants had been diagnosed with rheumatoid arthritis. It would be expedient to conduct this kind of studies with samples that include other patients with chronic pain (e.g., low back pain, fibromyalgia, etc.), in order to compare the results. A further experimental limitation involves the absence of a control group, and future studies are needed to replicate this study with a control group that can lead to a better understanding of the impact that variables such as age or cognitive anxiety have on attentional processes in a context far from pain. This study did not involve a preselection of the participants depending on the different variables analysed in it. For example, future researchers might select a sample with a high level of cognitive anxiety towards pain or confirm beforehand the presence of attentional bias in the participants. Finally, although it is true that the characteristics of the experimental task used here render it appropriate for forcing the appearance of the attentional bias [73], recent reviews of meta-analysis report that dot-probe has experimental limitations related to the use of verbal stimuli, whereby the use of other image-based experimental tasks is recommended [73, 76]. It is therefore advisable to bear these recommendations in mind for future research designs.

To conclude, most of the research that has studied attentional processes in patients with chronic pain has focused on understanding how attention and focalisation processes affect emotional variables. Nevertheless, and despite their limitations, the results presented here indicate the importance of having a more profound understanding of the impact that emotional variables and other variables such as age have on attentional processes and the rating of pain. The shortage of research in this matter prompts us to call for the need to conduct other studies to better understand the relationship between these variables. This information may be of considerable importance in the future, as it might help to provide better care for these patients.

## Figures and Tables

**Table 1:** List of words used in the dot-probe test.

Pain		Neutral
Dolorido–sore		Esponja–sponge
Golpes-blows		Dirección–address
Abrasador-searing		Pintado–painted
Angustiante–distressing		Timbre–bell
Alambre-wire		Calambres–cramp
Aplastante–overwhelming		Sótano–cellar
Sin aliento–breathless		Manopla–oven glove
Carcomer–undermine		Periódico–newspaper
Quejarse–complain		Tejado–roof
Daño–harm		Cuenco–bowl
Hiriente–hurtful		Vaso–glass
Inflamado–inflamed		Adorno–decoration
Nauseante–nauseating		Inodoro–toilet
Entumecido–swollen		Vidrio–window pane
Dolor–pain		Habitación–room
Penetrante-penetrating		Manto–blanket
Desgarrador		Estante–shelf
Pellizcar–pinch		Estufa–stove
Golpes-blows		Armario–cupboard
Pulsante–throbbing		Aparador–dresser
Agudo–sharp		Placa–plate
Punzante–stabbing		Lustre–shine
Inflamado-inflamed		Clavijas–pins
Espasmódico–spasmodic		Azulejos–tiles
Temible–frightening		Sacacorchos–corkscrew
Opresor-oppressive		Vecino-neighbour
Pinchazo–shooting		Barandilla-
Punzante-stabbing		Escobilla–brush
Desgarra–piercing		Imagen–image
Sensible–sensitive		Comida–food
Estremecimiento-shudder		Papel–paper
Tirante–tight		Microondas–microwave

**Table 2:** Sociodemographic characteristics, clinical variables, and relevant questionnaire scores of participants.

Characteristic	*n* = 52
Sex	
Female	39 (75)
Male	13 (25)

Age in years *(M, SD)*	54.7 ± (10.9)

Marital status	
Single	13 (25)
Married	28 (53.8)
Widowed	3 (5.8)
Divorced	4 (7.7)
Separated	4 (7.7)

Education level	
Primary	7 (13.5)
EGB or equivalent	7 (13.5)
Technical and vocational	11 (21.2)
Senior high school	12 (23.1)
University	11 (21.2)
Higher education	2 (3.8)
Unregulated studies	2 (3.8)

Employment status	
Service sector	8 (15.4)
Administrative services jobs	7 (13.5)
Professional or technician	6 (11.5)
Housewife	2 (3.8)
Student	2 (3.8)
Retired	13 (25)
Armed forces professionals	1 (1.9)
Unemployed	6 (11.5)
Others	7 (13.5)

Socioeconomic status	
Low	12 (23.1)
Medium	34 (65.4)
High	6 (11.5)

Diagnosis	
Rheumatoid arthritis	49 (94.2)
Low back pain	2 (3.8)
Fibromyalgia	1 (1.9)

Time elapsed since the first medical diagnosis	
Less than a year	2 (3.8)
Less than three years	5 (9.6)
Less than five years	2 (3.8)
Between five and ten years	14 (26.9)
More than 10 years	29 (55.8)

Total BDI-II short form score *(M, SD)*	4.21 ± (4.5)

McGill total PRI score *(M, SD)*	22.19 ± (8.3)

PASS-20 scale of cognitive anxiety *(M, SD)*	12.96 ± (4.4)

Values represent percentage (number) or mean ± standard deviation.

**Table 3:** Stepwise multiple regression analysis of the scales of the PRI on the mistakes made in the attention test.

Predictors	Unstandardised B	Standardised beta	Partial correlation
Constant	4.113^*∗∗*^		
Total PRI	−0.100	−0.285^*∗*^	−0.285

*R* = 0.285		*F* = 4.430^*∗∗*^	
*R* ^2^ = 0.81		Change of *R*^2^ = 0.81	
Adjusted *R*^2^ = 0.63		F Change = 4.430^*∗∗*^	

*N* = 52. ^*∗*^*p* < 0.05; ^*∗∗*^*p* < 0.01; ^*∗∗∗*^*p* < 0.001. *B* : unstandardised regression coefficient. Beta : standardised regression coefficient. Total PRI: scales of the pain rating index measured through the McGill Pain Questionnaire.

**Table 4:** Stepwise multiple regression analysis of depression, cognitive anxiety towards pain, the scales of the PRI, and age over total performance times.

Predictors	Unstandardised B	Standardised beta	Partial correlation
Constant	52966.252^*∗∗*^		
Age	1039.866	0.405^*∗∗*^	0.429
Cognitive anxiety	−1954.536	−0.310^*∗∗*^	−0.341

*R* = 0.521		*F* = 9.116^*∗∗∗*^	
*R* ^2^ = 0.271		Change of *R*^2^ = 0.096	
Adjusted *R*^2^ = 0.241		F change = 6.467^*∗*^	

*N* = 52 ^*∗*^*p* < 0.05; ^*∗∗*^*p* < 0.01; ^*∗∗∗*^*p* < 0.001. *B* : unstandardised regression coefficient. Beta : standardised regression coefficient. Age: participants' age in years. Cognitive anxiety: scale of cognitive anxiety towards pain measured by PASS-20.

## Data Availability

The data used to support the findings of this study have been deposited as an attachment with the following name “fulldatabase-art1-en.sav”, as supplementary information accompanying this paper.

## Abbreviations

MBI: Mindfulness-based intervention.
CBT: Cognitive behavioural therapy.

## References

[1] Català E., Reig E., Artés M., Aliaga L., López J. S., Segú J. L. (2002). Prevalence of pain in the Spanish population telephone survey in 5000 homes. *European Journal of Pain*.

[2] Breivik H., Collett B., Ventafridda V., Cohen R., Gallacher D. (2006). Survey of chronic pain in Europe: prevalence, impact on daily life, and treatment. *European Journal of Pain*.

[3] Langley P. C., Ruiz-Iban M. A., Molina J. T., de Andres J., Castellón J. R. G.-E. (2011). The prevalence, correlates and treatment of pain in Spain. *Journal of Medical Economics*.

[4] Phillips C. J. (2014). Economic burden of chronic pain. *Expert Review of Pharmacoeconomics & Outcomes Research*.

[5] Melzack R., Wall P. D. (1965). Pain mechanisms: a new theory. *Science*.

[6] Melzack R., Casey K. L., Kenshalo D. (1968). Sensory, motivational, and central control determinants of pain. A new conceptual model. *The Skin Senses*.

[7] Bair M. J., Wu J., Damush T. M., Sutherland J. M., Kroenke K. (2008). Association of depression and anxiety alone and in combination with chronic musculoskeletal pain in primary care patients. *Psychosomatic Medicine*.

[8] Casado M. I., Moix J., Vidal J. (2005). Etiología, cronificación y tratamiento el dolor lumbar. *Clínica Y Salud*.

[9] Casado M. I., Urbano M. P. (2001). Emociones negativas y dolor crónico. *Ansiedad Y Estrés*.

[10] Moix J., Casado M. I. (2011). Terapias Psicológicas para el Tratamiento del Dolor Crónico. *Clínica Y Salud*.

[11] Mok L. C., Lee I. F.-K. (2008). Anxiety, depression and pain intensity in patients with low back pain who are admitted to acute care hospitals. *Journal of Clinical Nursing*.

[12] Waxman S. E., Tripp D. A., Flamenbaum R. (2008). The mediating role of depression and negative partner responses in chronic low back pain and relationship satisfaction. *The Journal of Pain*.

[13] Morley S., Shapiro D. A., Biggs J. (2004). Developing a treatment manual for attention management in chronic pain. *Cognitive Behaviour Therapy*.

[14] Redondo M. M., León Mateos L., Pérez Nieto M. A., Jover Jover J. A., Abasolo Alcázar L. (2008). El dolor en los pacientes con artritis reumatoide: variables psicológicas relacionadas e intervención. *Clínica Y Salud*.

[15] Ahles T. A., Blanchard E. B., Leventhal H. (1983). Cognitive control of pain: attention to the sensory aspects of the cold pressor stimulus. *Cognitive Therapy and Research*.

[16] Cioffi D., Holloway J. (1993). Delayed costs of suppressed pain. *Journal of Personality and Social Psychology*.

[17] Dar R., Leventhal H. (1993). Schematic processes in pain perception. *Cognitive Therapy and Research*.

[18] Gilligan R. M., Ascher L. M., Wolper J., Bochachevsky C. (1984). Comparison of three cognitive strategies in altering pain behaviors on a cold pressor task. *Perceptual and Motor Skills*.

[19] Nouwen A., Cloutier C., Kappas A., Warbrick T., Sheffield D. (2006). Effects of focusing and distraction on cold pressor-induced pain in chronic back pain patients and control subjects. *The Journal of Pain*.

[20] Tracey I., Ploghaus A., Gati J. S. (2002). Imaging attentional modulation of pain in the periaqueductal gray in humans. *Journal of Neuroscience*.

[21] Edwards R. R., Bingham C. O., Bathon J., Haythornthwaite J. A. (2006). Catastrophizing and pain in arthritis, fibromyalgia, and other rheumatic diseases. *Arthritis & Rheumatism*.

[22] Edwards R. R., Cahalan C., Mensing G., Smith G., Haythornthwaite J. A. (2006). Pain, catastrophizing, and depression in the rheumatic diseases. *Pain*.

[23] He C.-H., Yu F., Jiang Z.-C., Wang J.-Y., Luo F. (2014). Fearful thinking predicts hypervigilance towards pain-related stimuli in patients with chronic pain. *PsyCh Journal*.

[24] Leeuw M., Goossens M. E. J. B., Linton S. J., Crombez G., Boersma K., Vlaeyen J. W. S. (2007). The fear-avoidance model of musculoskeletal pain: current state of scientific evidence. *Journal of Behavioral Medicine*.

[25] Gagliese L., Melzack R. (1997). Chronic pain in elderly people. *Pain*.

[26] Gallagher R. M., Verma S., Mossey J. (2000). Chronic pain. Sources of late-life pain and risk factors for disability. *Geriatrics*.

[27] Jorge L. L., Gerard C., Revel M. (2009). Evidences of memory dysfunction and maladaptive coping in chronic low back pain and rheumatoid arthritis patients: challenges for rehabilitation. *European Journal of Physical and Rehabilitation Medicine*.

[28] Oosterman J. M., Derksen L. C., van Wijck A. J., Veldhuijzen D. S., Kessels R. P. C. (2011). Memory functions in chronic pain. *The Clinical Journal of Pain*.

[29] Oosterman J. M., Gibson S. J., Pulles W. L. J. A., Veldhuijzen D. S. (2013). On the moderating role of age in the relationship between pain and cognition. *European Journal of Pain*.

[30] Moriarty O., Ruane N., O’Gorman D. (2017). Cognitive impairment in patients with chronic neuropathic or radicular pain: an interaction of pain and age. *Frontiers in Behavioral Neuroscience*.

[31] Moriarty O., Joukje M., van Wijck A. J. (2012). Executive and attentional functions in chronic pain: does performance decrease with increasing task load?. *Pain Research and Management*.

[32] Ehde D. M., Dillworth T. M., Turner J. A. (2014). Cognitive-behavioral therapy for individuals with chronic pain: efficacy, innovations, and directions for research. *American Psychologist*.

[33] Åkerblom S., Perrin S., Rivano Fischer M., McCracken L. M. (2015). The mediating role of acceptance in multidisciplinary cognitive-behavioral therapy for chronic pain. *The Journal of Pain*.

[34] Leventhal H. (1992). I know distraction works even though it doesn’t!. *Health Psychology*.

[35] Turk D. C., Meichenbaum D. H., Wall P. D., Melzack R. (1994). A cognitive-behavioral approach to pain management. *Textbook of Pain*.

[36] Arntz A., Dreessen L., De Jong P. (1994). The influence of anxiety on pain: attentional and attributional mediators. *Pain*.

[37] Eccleston C., Crombez G. (1999). Pain demands attention: a cognitive-affective model of the interruptive function of pain. *Psychological Bulletin*.

[38] Farthing G. W., Venturino M., Brown S. W. (1984). Suggestion and distraction in the control of pain: test of two hypotheses. *Journal of Abnormal Psychology*.

[39] Fernandez E., Turk D. C. (1989). The utility of cognitive coping strategies for altering pain perception: a meta-analysis. *Pain*.

[40] Miltner W., Johnson R., Braun C., Larbig W. (1989). Somatosensory event-related potentials to painful and non-painful stimuli: effects of attention. *Pain*.

[41] McCaul K. D., Malott J. M. (1984). Distraction and coping with pain. *Psychological Bulletin*.

[42] Keefe F. J., Williams D. A. (1990). A comparison of coping strategies in chronic pain patients in different age groups. *Journal of Gerontology*.

[43] Lautenbacher S., Huber C., Schöfer D. (2010). Attentional and emotional mechanisms related to pain as predictors of chronic postoperative pain: a comparison with other psychological and physiological predictors. *Pain*.

[44] McCaul K. D., Monson N., Maki R. H. (1992). Does distraction reduce pain-produced distress among college students?. *Health Psychology*.

[45] Todd J., Sharpe L., Colagiuri B., Khatibi A. (2016). The effect of threat on cognitive biases and pain outcomes: an eye-tracking study. *European Journal of Pain*.

[46] Turner J. A., Clancy S. (1986). Strategies for coping with chronic low back pain: relationship to pain and disability. *Pain*.

[47] Asmundson G. J., Norton P. J., Vlaeyen J. W. (2004). *Fear-avoidance Models of Chronic Pain: An Overview, Understanding and Treating Fear of Pain*.

[48] Vlaeyen J. W. S., Linton S. J. (2000). Fear-avoidance and its consequences in chronic musculoskeletal pain: a state of the art. *Pain*.

[49] McCracken L. M. (2005). *Contextual Cognitive-Behavioral Therapy For Chronic Pain*.

[50] Vowles K. E., McCracken L. M. (2008). Acceptance and values-based action in chronic pain: a study of treatment effectiveness and process. *Journal of Consulting and Clinical Psychology*.

[51] Crombez G., Eccleston C., Van Damme S., Vlaeyen J. W. S., Karoly P. (2012). Fear-avoidance model of chronic pain. *The Clinical Journal oF Pain*.

[52] Siegel S. (2005). Drug tolerance, drug addiction, and drug anticipation. *Current Directions in Psychological Science*.

[53] Sharp T. J. (2001). Chronic pain: a reformulation of the cognitive-behavioural model. *Behaviour Research and Therapy*.

[54] Asmundson G. J. G., Norton P. J., Norton G. R. (1999). Beyond pain. *Clinical Psychology Review*.

[55] Crombez G., Eccleston C., Baeyens F., Eelen P. (1998). When somatic information threatens, catastrophic thinking enhances attentional interference. *Pain*.

[56] Crombez G., Vlaeyen J. W., Heuts P. H., Lysens R. (1999). Pain-related fear is more disabling than pain itself: evidence on the role of pain-related fear in chronic back pain disability. *Pain*.

[57] Linton S. J. (2005). Do psychological factors increase the risk for back pain in the general population in both a cross-sectional and prospective analysis?. *European Journal of Pain*.

[58] McCracken L. M., Samuel V. M. (2007). The role of avoidance, pacing, and other activity patterns in chronic pain. *Pain*.

[59] Vangronsveld K., Peters M., Goossens M., Linton S., Vlaeyen J. (2007). Applying the fear-avoidance model to the chronic whiplash syndrome. *Pain*.

[60] Vangronsveld K. L. H., Peters M., Goossens M., Vlaeyen J. (2008). The influence of fear of movement and pain catastrophizing on daily pain and disability in individuals with acute whiplash injury: a daily diary study. *Pain*.

[61] Vlaeyen J. W. S., Morley S., Crombez G. (2016). The experimental analysis of the interruptive, interfering, and identity-distorting effects of chronic pain. *Behaviour Research and Therapy*.

[62] Hervás G., Cebolla A., Soler J. (2016). Intervenciones psicológicas basadas en mindfulness y sus beneficios: estado actual de la cuestión. *Clínica Y Salud*.

[63] Hayes A., Feldman G. (2004). Clarifying the construct of mindfulness in the context of emotion regulation and the process of change in therapy. *Clinical Psychology: Science and Practice*.

[64] McCracken L. M., Gauntlett-Gilbert J., Vowles K. E. (2007). The role of mindfulness in a contextual cognitive-behavioral analysis of chronic pain-related suffering and disability. *Pain*.

[65] Bishop S. R., Lau M., Shapiro S. (2004). Mindfulness: a proposed operational definition. *Clinical Psychology: Science and Practice*.

[66] Kabat-Zinn J. (1982). An outpatient program in behavioral medicine for chronic pain patients based on the practice of mindfulness meditation: theoretical considerations and preliminary results. *General Hospital Psychiatry*.

[67] Palao A., Torrijos M., Del Río M., Muñoz-Sanjosé A., Rodríguez B. (2019). Intervenciones basadas en mindfulness y compasión en dolor crónico. *Facultad de Medicina y Ciencias de la Salud UAH*.

[68] Quintana M., Rincón M. E. (2011). Eficacia del entrenamiento mindfulness para pacientes con fibromialgia. *Clínica Y Salud*.

[69] Hayes S. C., Strosahl K. D., Wilson K. G. (1994). *Terapia de Aceptación y Compromiso. Proceso y práctica del cambio consciente (Mindfulness)*.

[70] Wetherell J. L., Afari N., Rutledge T. (2011). A randomized, controlled trial of acceptance and commitment therapy and cognitive-behavioral therapy for chronic pain. *Pain*.

[71] Veehof M. M., Oskam M.-J., Schreurs K. M. G., Bohlmeijer E. T. (2011). Acceptance-based interventions for the treatment of chronic pain: a systematic review and meta-analysis. *Pain*.

[72] Casey K. L., Lorenz J. (2000). The determinants of pain revisited: coordinates in sensory space. *Pain Research and Management*.

[73] Crombez G., Van Ryckeghem D. M. L., Eccleston C., Van Damme S. (2013). Attentional bias to pain-related information: a meta-analysis. *Pain*.

[74] MacLeod C., Mathews A., Tata P. (1986). Attentional bias in emotional disorders. *Journal of Abnormal Psychology*.

[75] Todd J., Sharpe L., Johnson A., Nicholson Perry K., Colagiuri B., Dear B. F. (2015). Towards a new model of attentional biases in the development, maintenance, and management of pain. *Pain*.

[76] Todd J., van Ryckeghem D. M. L., Sharpe L., Crombez G. (2018). Attentional bias to pain-related information: a meta-analysis of dot-probe studies. *Health Psychology Review*.

[77] Beck A. T., Steer R. A., Brown G. K. (1996). Beck depression inventory-II. *San Antonio*.

[78] Sanz J., García-Vera M. P., Fortún M., Espinosa R. (2005). Desarrollo y propiedades psicométricas de una versión breve española del inventario para la depresión de Beck-II (BDI-II). *Comunicación presentada en el V Congreso Iberoamericano de Evaluación Psicológica*.

[79] Prieto G., Muñiz J. (2000). Un modelo para evaluar la calidad de los tests utilizados en España. *Papeles del psicólogo*.

[80] Melzack R. (1975). The McGill Pain Questionnaire: major properties and scoring methods. *Pain*.

[81] Lazaro C., Bosch F., Torrubia R., Baños J. E. (1994). The development of a Spanish questionnaire for assessing pain: preliminary data concerning reliability and validity. *Europe Journal Psychological Assessment*.

[82] McCracken L. M., Dhingra L. (2002). A short version of the Pain Anxiety Symptoms Scale (PASS-20): preliminary development and validity. *Pain Research and Management*.

[83] Haggman S. P., Sharpe L. A., Nicholas M. K., Refshauge K. M. (2010). Attentional biases toward sensory pain words in acute and chronic pain patients. *The Journal of Pain*.

[84] Keogh E., Ellery D., Hunt C., Hannent I. (2001). Selective attentional bias for pain-related stimuli amongst pain fearful individuals. *Pain*.

[85] Posner M. I. (1980). Orienting of attention. *Quarterly Journal of Experimental Psychology*.

[86] Cohen J. (1988). *Statistical Power Analysis for the Behavioral Sciences*.

[87] Pearce S. A., Isherwood S., Hrouda D., Richardson P. H., Erskine A., Skinner J. (1990). Memory and pain: tests of mood congruity and state dependent learning in experimentally induced and clinical pain. *Pain*.

[88] Crombez G., Hermans D., Adriaensen H. (2000). The emotional stroop task and chronic pain: what is threatening for chronic pain sufferers?. *European Journal of Pain*.

[89] Dehghani M., Sharpe L., Nicholas M. K. (2003). Selective attention to pain-related information in chronic musculoskeletal pain patients. *Pain*.

[90] Duschek S., Werner N. S., Limbert N., Winkelmann A., Montoya P. (2014). Attentional bias toward negative information in patients with fibromyalgia syndrome. *Pain*.

[91] Pearce J., Morley S. (1989). An experimental investigation of the construct validity of the McGill Pain Questionnaire. *Pain*.

[92] Sharpe L., Dear B. F., Schrieber L. (2009). Attentional biases in chronic pain associated with rheumatoid arthritis: hypervigilance or difficulties disengaging?. *The Journal of Pain*.

[93] Snider B. S., Asmundson G. J. G., Wiese K. C. (2000). Automatic and strategic processing of threat cues in patients with chronic pain: a modified stroop evaluation. *The Clinical Journal of Pain*.

[94] Fashler S., Katz J. (2014). More than meets the eye: visual attention biases in individuals reporting chronic pain. *Journal of Pain Research*.

[95] Khatibi A., Dehghani M., Sharpe L., Asmundson G. J., Pouretemad H. (2009). Selective attention towards painful faces among chronic pain patients: evidence from a modified version of the dot-probe. *Pain*.

[96] Schoth D. E., Nunes V. D., Liossi C. (2012). Attentional bias towards pain-related information in chronic pain; a meta-analysis of visual-probe investigations. *Clinical Psychology Review*.

[97] Yang Z., Jackson T., Chen H. (2013). Effects of chronic pain and pain-related fear on orienting and maintenance of attention: an eye movement study. *The Journal of Pain*.

[98] Andersson G., Haldrup D. (2003). Personalized pain words and stroop interference in chronic pain patients. *European Journal of Pain*.

[99] Asmundson G. J. G., Wright K. D., Hadjistavropoulos H. D. (2005). Hypervigilance and attentional fixedness in chronic musculoskeletal pain: consistency of findings across modified stroop and dot-probe tasks. *The Journal of Pain*.

[100] Pincus T., Fraser L., Pearce S. (1998). Do chronic pain patients ’stroop’ on pain stimuli?. *British Journal of Clinical Psychology*.

[101] LeBlanc J., Potvin P. (1966). Studies on habituation to cold pain. *Canadian Journal of Physiology and Pharmacology*.

[102] Rennefeld C., Wiech K., Schoell E. D., Lorenz J., Bingel U. (2010). Habituation to pain: further support for a central component. *Pain*.

[103] Strempel H. (1976). Adaptive Modifikationen des Klteschmerzes. *European Journal of Applied Physiology and Occupational Physiology*.

[104] Strempel H. (1978). Adaptive Modifikationen des KÄlteschmerzes III. Mitteilung: kurzzeitversuche mit 1-Min-Intervallen. *European Journal of Applied Physiology and Occupational Physiology*.

[105] Eccleston C. (1994). Chronic pain and attention: a cognitive approach. *British Journal of Clinical Psychology*.

[106] Sánchez I. Y., Pérez V. T. (2008). El funcionamiento cognitivo en la vejez: atención y percepción en el adulto mayor. *Revista Cubana de Medicina General Integral*.

[107] Fernández-Ballesteros R. (2000). *Gerontología Social*.

[108] Eysenck M. W. (1982). *Attention and Arousal: Cognition and Performance*.

[109] Deffenbacher J. L., Sarason I. G. (1980). Worry and emotionality in test anxiety. *Test Anxiety: Theory, Research and Application*.

[110] Morris L. W., Davis M. A., Hutchings C. H. (1981). Cognitive and emotional components of anxiety: literature review and a revised worry-emotionality scale. *Journal of Educational Psychology*.

[111] Eysenck M. W. (1979). Anxiety, learning, and memory: a reconceptualization. *Journal of Research in Personality*.

[112] Eysenck M. W., Calvo M. G. (1992). Anxiety and performance: the processing efficiency theory. *Cognition & Emotion*.

[113] Mogg K., Bradley B. P., Hallowell N., Macgregor-Morris R. (1994). Attentional bias to threat: roles of trait anxiety, stressful events, and awareness. *The Quarterly Journal of Experimental Psychology Section A*.

[114] Mogg K., Mathews A., Bird C., Macgregor-Morris R. (1990). Effects of stress and anxiety on the processing of threat stimuli. *Journal of Personality and Social Psychology*.

[115] Fox E., Russo R., Bowles R., Dutton K. (2001). Do threatening stimuli draw or hold visual attention in subclinical anxiety?. *Journal of Experimental Psychology: General*.

[116] García-Nieto R., Ortega-Ladrón de Cegama E., Ruiz de Santos E., Lorenzo J. M. (2008). Déficit de memoria en una muestra de pacientes con dolor crónico. *Revista de la Sociedad Española del Dolor*.

[117] Douglas W., Graham C., Anderson D., Rogerson K. (2004). Managing chronic pain through cognitive change and multidisciplinary treatment program. *Australian Psychologist*.

